# An Outside-the-Box Approach for Treating a Rare Case of Urethrovaginal Fistula

**DOI:** 10.7759/cureus.74614

**Published:** 2024-11-27

**Authors:** Rahul Agrawal, Sankalp Goel, Vilas P Sabale, Vikram Satav

**Affiliations:** 1 Urology, Dr. D. Y. Patil Medical College, Hospital and Research Centre, Dr. D. Y. Patil Vidyapeeth (Deemed to be University) Pune, Pune, IND; 2 Plastic Surgery, Dr. D. Y. Patil Medical College, Hospital and Research Centre, Dr. D. Y. Patil Vidyapeeth (Deemed to be University) Pune, Pune, IND

**Keywords:** fistula repair, gynaecology, matriderm, urethrovaginal fistula, urology

## Abstract

Urethrovaginal fistulas are rare complications often arising from urethrovaginal injuries commonly due to obstetrical trauma, urethral surgeries, pelvic fractures, or neoplastic treatments. Here, we present a unique case involving a 23-year-old female patient with a large urethrovaginal fistula and complete anterior vaginal wall sloughing following prolonged obstructed labor. Nine months post-cesarean, she reported urine leakage via the vagina upon catheter removal, which intensified in an erect posture. Clinical examination revealed an absent urethra and a wide bladder neck with the posterior bladder wall visible through the vaginal introitus, indicating severe tissue loss. Following interdisciplinary consultations, a novel reconstructive surgery was planned under urology. During surgery, the bladder and ureters were safeguarded using double J (DJ) stents, and a neo-urethral tube was fashioned from a strip of the anterior vaginal wall. The bladder neck was narrowed, and artificial dermal collagen (Matriderm®) was applied as an interpositional waterproof layer, representing an innovation previously undocumented in similar cases. Postoperative recovery was uneventful, and after catheter removal, the patient regained continence and normal urinary function. This case suggests a potential role for dermal substitutes in urological reconstructions, particularly in cases requiring waterproof tissue closures, which warrants further investigation.

## Introduction

Urethrovaginal fistula is a rare condition that typically arises from urethrovaginal injury. Common causes include obstetric trauma, anterior vaginal repairs, urethral diverticulectomy, pelvic fractures, and vaginal or urethral neoplasms, particularly when associated with radiation therapy [1]. Symptoms vary depending on the fistula’s location relative to the sphincter mechanism. Proximal fistulas often result in continuous or stress incontinence, whereas distal fistulas may be asymptomatic or lead to messy voiding [2,3]. Pseudoincontinence can occur when urine passes through the fistula into the vagina, subsequently emptying when the patient stands. Here, we present a unique case of a wide urethrovaginal fistula with complete sloughing of the anterior vaginal wall, managed using an innovative and unconventional approach.

## Case presentation

History

A 23-year-old woman presented to our department with chief complaints of leakage of urine per vagina for nine (A1) months. The patient had a history of obstructed labor nine months prior, which required lower segment cesarean section (LSCS) for the delivery. On postoperative day three, after the removal of the urinary catheter, the patient noticed urinary leaking per her vagina, which would increase in an erect position. The patient had no other co-morbidities and no other significant history. She reported to us nine months after delivery.

Examination

**Figure 1:**
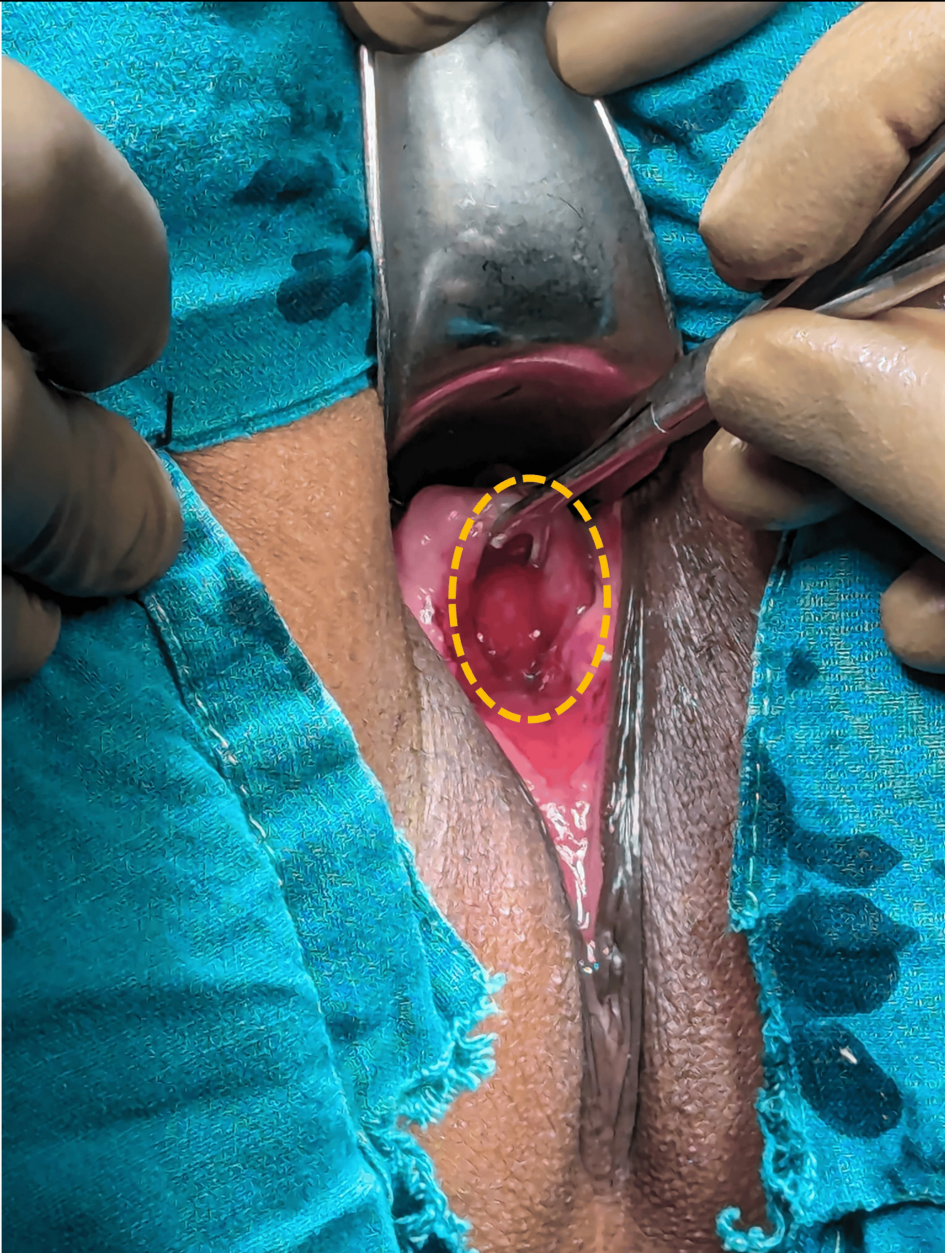
With the patient in jackknife position, the fistula (enclosed yellow circle) can be visualized through which the posterior bladder wall can be visualized.

A per vaginal examination revealed a defect in the anterior vaginal wall, large enough to admit two fingers. The margins of the defect were indistinct, giving the impression of a single cavity. Visualization through a speculum examination revealed the posterior wall of the bladder, as shown in Figure 1, where the yellow dotted line highlights the defect through which the bladder's posterior wall is visible. This presentation was likely due to sloughing of the posterior urethral wall and the anterior vaginal wall, extending from the bladder neck to the external urinary meatus. A bladder function test was deemed unnecessary, as the patient was completely incontinent.

Planning

To address the fistula, an interdisciplinary meeting was held, involving specialists from urology, plastic and reconstructive surgery, and gynecology. It was determined that the two cavities needed to be separated with the interposition of soft tissue, as per standard practice. However, due to the size of the defect, the gold-standard Martius flap was deemed too small to adequately cover the area. Muscle flap options, such as gracilis and rectus abdominis muscles, were considered for reliable coverage. However, upon discussing these options with the patient, she expressed her preference to avoid additional scars on her abdomen or thigh and requested an alternative method for fistula closure.

The consulting plastic surgeon proposed a novel approach involving the use of an acellular dermal matrix as interpositional tissue between the two cavities. The rationale behind this approach was based on the dermal template's ability to acquire vascularization from surrounding tissues and form a neodermis. A review of the literature revealed no prior research on this technique. However, once the dermal matrix becomes vascularized, it essentially functions as a vascularized tissue, mimicking the properties of a flap [4]. This plan was explained to the patient, with an emphasis on the experimental nature of the procedure. The patient provided informed consent for the procedure and also agreed to the publication of the technique and related photographs for educational purposes.

Surgical steps

The patient first underwent a cystoscopy in the lithotomy position, which confirmed the per vaginal findings. The bladder was found to be normal with both ureteric openings away from the fistula. Double J (DJ) stents were placed bilaterally to safeguard the ureteric openings and intramural tunnel during dissection and reconstruction. The patient was then kept in a jackknife position. A 2.5-cm strip of anterior vaginal wall was marked to create a urethral tube (akin to male second-stage urethroplasty), and the same incision was extended around the bladder neck.

**Figure 2:**
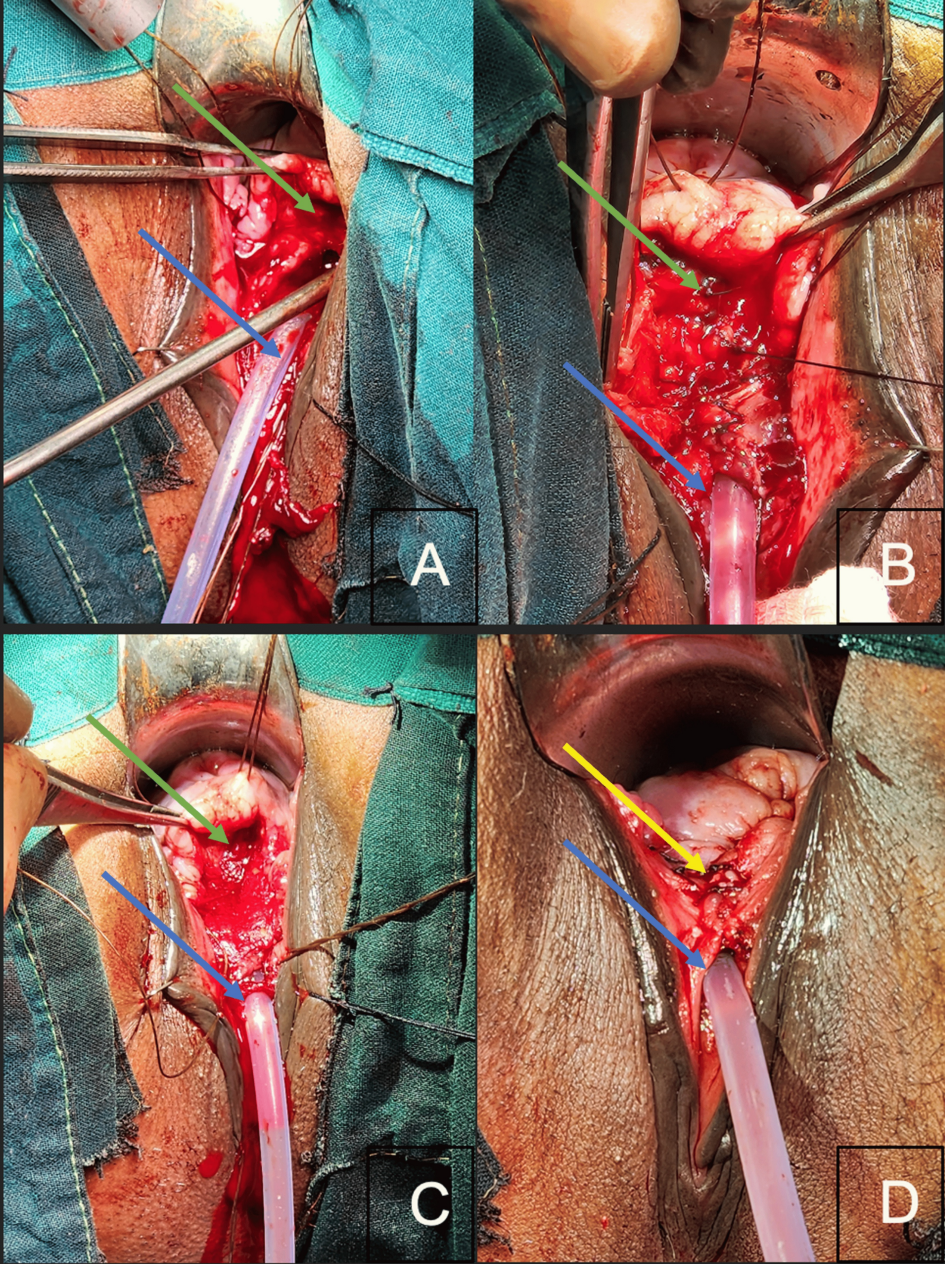
Intra-operative photos depicting (A) closure of the urethra (green arrow) over the silicon Foley's catheter (blue arrow); (B) closure of the vagina’s anterior wall (green arrow); (C) application of Matriderm® in between the suture lines of both cavities; and (D) complete closure of the urethrovaginal fistula (yellow arrow). Green arrow: urethrovaginal fistula, blue arrow: foley's catheter, and yellow arrow: closed urethrovaginal fistula.

A plane between the anterior vaginal wall and bladder was dissected toward the anterior fornix, creating a satisfactorily mobile anterior vaginal wall flap while safeguarding the ureteric orifices and the intramural tunnel (particularly on the right side), as guided by the DJ stents. The bladder neck was narrowed, as shown in Figure 2A, and a urethral tube was created over a 16Fr silicone Foley catheter, forming a neo-urethra, neo-meatus, and neo-bladder neck, as illustrated in Figure 2B. For the second layer, an artificial dermal matrix, Matriderm® (1 mm thickness, fenestrated, 52 mm x 27 mm), was used as an interposition flap. To maintain separation between the repaired urethra and vaginal wall, Matriderm® was placed over the repaired urethra and moistened with normal saline, as seen in Figure 2C. The anterior vaginal flap was then advanced to cover the newly formed urethra and Matriderm® complex, as depicted in Figure 2D. The postoperative period was uneventful.

Post-operative follow-up

**Figure 3:**
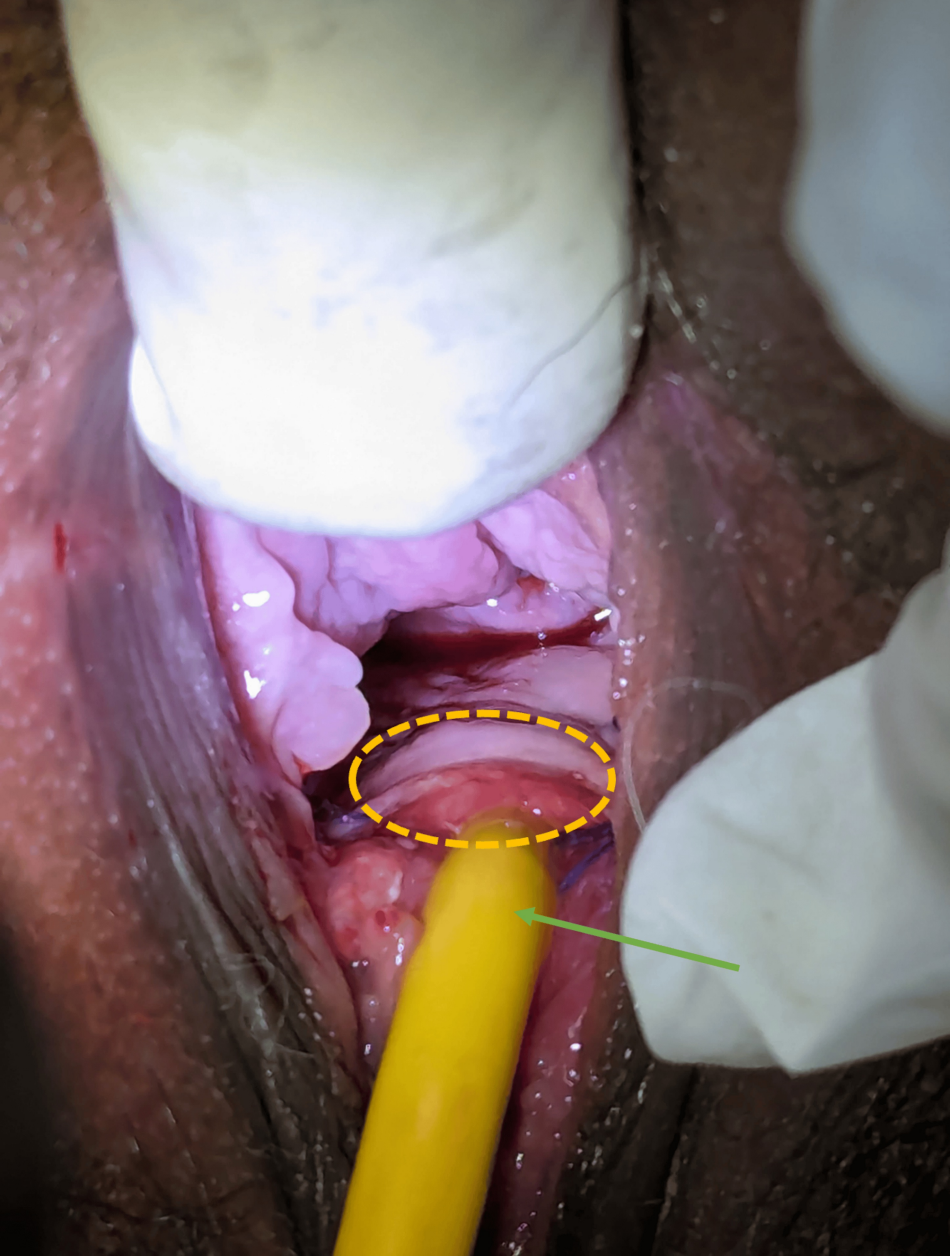
Post-operative image of the well-healed and continent urethra and vagina, closed fistula (circle), and Foley’s catheter (green arrow).

After three weeks, the patient presented with the catheter in situ, as shown in Figure 3. There were no complaints of incontinence, peri-catheter leakage, pain, or discharge. On examination, the suture line was healthy, with clear separation of the two repaired cavities. There was no odor or evidence of leakage. The catheter was removed, and the patient was completely continent, voiding regularly and fully, without residual urine. At the six-week follow-up, the patient’s DJ stent was removed using a cystoscope, which passed easily through the repaired urethra. At the three-month follow-up, the patient was satisfied, dry, continent, and free of odor.

## Discussion

Even in 2024, adequate and prompt obstetric care is still far from adequate in the Indian subcontinent [2]. This is one such case of obstructed labor that resulted in complete sloughing of the anterior vaginal wall, posterior urethral wall, and the bladder neck to a certain extent, which resulted in a wide-open bladder neck freely draining into the vagina.

Management of urethrovaginal fistula depends on a variety of factors, namely the presence or absence of symptoms, etiology, and the size and location of the fistula. Surgical procedures that have been described include urethral marsupialization, vaginal flap closure, labial fat pad repair (Martius procedure), full-thickness skin graft reconstruction, musculocutaneous flap interposition, and bladder flap techniques [3,5-7].

In this case, the management involved collaboration between a gynecologist and reconstructive surgeons following a discussion of various treatment options. The Martius flap was deemed unlikely to provide adequate coverage. Although an intervening layer of the rectus abdominis flap was present, it could not be utilized due to the patient’s lower segment cesarean section (LSCS) scar and the potential transection of the rectus abdominis. Additionally, the patient did not consent to the use of other viable options, such as gracilis muscle transposition.

In this case, the management involved collaboration between a gynecologist and reconstructive surgeons following a discussion of various treatment options. The Martius flap was deemed unlikely to provide adequate coverage. Although an intervening layer of the rectus abdominis flap was present, it could not be utilized due to the patient’s lower segment cesarean section (LSCS) scar and the potential transection of the rectus abdominis. Additionally, the patient did not consent to the use of other viable options, such as gracilis muscle transposition.

Due to the unavailability of autologous interposition flaps, a relatively new dermal substitute containing a collagen and elastin matrix (Matriderm®) was considered for use as a scaffold. While other dermal substitutes are available in the market, Matriderm® offers the advantage of being used in a single-stage reconstruction procedure, as it lacks a protective removable layer. In contrast, other matrices typically require a two-step process, including the removal of the protective layer after matrix integration. Following consultation with reconstructive surgeons, Matriderm® was utilized to cover the suture line and raw surfaces. Its mechanism of action involves invading cells that use the matrix fibers as guiding ridges for structured healing. These cells recognize binding sites on the native collagen fibers, which activates them upon binding. The activated fibroblasts subsequently produce the body’s own collagen [8-10].

We counseled the patient regarding the use of the dermal template as an experimental procedure, which had not been performed before, and the patient provided informed consent. The patient was also informed about the potential for a variable degree of persistent incontinence, the possibility of requiring diversion, and the need for subsequent procedures, such as the Young-De Leede technique. However, following catheter removal, the patient demonstrated complete continence and satisfactory voiding at regular intervals.

Matriderm® is commonly used in wounds with poor vascular beds or those requiring increased dermal thickness, followed by the application of a split-thickness skin graft. Matriderm® provides a matrix that is rapidly infiltrated by fibroblasts and vascularized by capillary loops, forming tissue that supports and nourishes the overlying split-thickness skin graft. Over time, this tissue matures into a neo-dermis, offering stability and durability to the overlying skin layers [11]. However, a notable disadvantage of using this material is its relatively high cost compared to other reconstruction methods, as well as its limited availability in some regions.

## Conclusions

Urethrovaginal fistula is a rare condition that necessitates surgical intervention due to the significant social stigma and functional inconvenience associated with its symptoms. Surgeons have access to a wide array of techniques to address such defects. However, in cases where conventional approaches are not viable, alternative strategies must be explored. In this case, we employed a dermal substitute to create a waterproof barrier between tissues prone to leakage. The dermal substitute, supported by vascularization from surrounding tissue, acted as an interpositional layer between two structures prone to fistula recurrence. While it cannot be definitively established that the use of Matriderm® was solely responsible for the favorable outcome in this case, its contribution appears to have been significant. To the best of our knowledge, there are no prior reports on the use of Matriderm® as a tissue interposition material in such repairs. Further research is necessary to investigate additional applications of dermal substitutes in urology, including their potential use as stable tissue coverage for urethroplasty, ureteroplasty, hypospadias repair, epispadias correction, chordee correction, and other related procedures.
